# Accelerated tooth movement in *Rsk2*-deficient mice with impaired cementum formation

**DOI:** 10.1038/s41368-020-00102-4

**Published:** 2020-12-23

**Authors:** Cita Nottmeier, Maximilian G. Decker, Julia Luther, Simon von Kroge, Bärbel Kahl-Nieke, Michael Amling, Thorsten Schinke, Julian Petersen, Till Koehne

**Affiliations:** 1 Department of Orthodontics, University Medical Center Hamburg-Eppendorf, Hamburg, Germany; 2 Institute of Osteology and Biomechanics, University Medical Center Hamburg-Eppendorf, Hamburg, Germany

**Keywords:** Medical research, Risk factors, Cementum

## Abstract

Coffin–Lowry–Syndrome (CLS) is a X-linked mental retardation characterized by skeletal dysplasia and premature tooth loss. We and others have previously demonstrated that the ribosomal S6 kinase RSK2, mutated in CLS, is essential for bone and cementum formation; however, it remains to be established whether RSK2 plays also a role in mechanically induced bone remodeling during orthodontic tooth movement (OTM). We, therefore, performed OTM in wild-type (WT) mice and Rsk2-deficient mice using Nitinol tension springs that were fixed between the upper left molars and the incisors. The untreated contralateral molars served as internal controls. After 12 days of OTM, the jaws were removed and examined by micro-computed tomography (µCT), decalcified histology, and immunohistochemistry. Our analysis of the untreated teeth confirmed that the periodontal phenotype of *Rsk2*-deficient mice is characterized by alveolar bone loss and hypoplasia of root cementum. Quantification of OTM using µCT revealed that OTM was more than two-fold faster in *Rsk2*-deficient mice as compared to WT. We also observed that OTM caused alveolar bone loss and root resorptions in WT and *Rsk2*-deficient mice. However, quantification of these orthodontic side effects revealed no differences between WT and *Rsk2*-deficient mice. Taken together, *Rsk2* loss-of-function accelerates OTM in mice without causing more side effects.

## Introduction

The growth factor regulated ribosomal S6 kinase RSK2 (RPS6KA3) belongs to a group of widely expressed serine/threonine kinases, that are important for growth, survival, and proliferation of cells.^1–3^ RSK2 operates at the distal end of the Ras/Map kinase pathway and phosphorylates different substrates, such as cAMP response element-binding protein (CREB), c-Fos, activating transcription factor 4 (ATF4), and estrogen receptor alpha (ERα).^4–6^ CREB, ATF4, and ERα are known to induce differentiation of bone-forming osteoblasts,^4,7–12^ whereas c-Fos is required for differentiation of bone-resorbing osteoclasts.^13^ RSK2, therefore, plays a pivotal role in bone turnover.

The role of RSK2 in bone homeostasis was deciphered by skeletal analyses of humans with Coffin–Lowry syndrome (CLS), carrying inactivating mutations in the *Rsk2* gene, and by analyses of the corresponding *Rsk2*-deficient mouse model. Importantly, oral and dental findings in CLS patients also suggest a central role of RSK2 during dental development. The oral phenotype of CLS patients is characterized by hypodontia, microdontia, delayed eruption, and premature tooth loss.^14–17^ Employing *Rsk2*-deficient mice we have previously demonstrated that the premature tooth loss of CLS patients can be attributed to a cell-autonomous defect in cementoblast activity causing hypoplasia of root cementum, detachment of periodontal fibers, and subsequent alveolar bone loss.^18^ These findings were consistent with case reports describing that prematurely lost teeth of CLS patients had a reduced layer of root cementum.^14,16^

Despite its key function for bone and periodontal homeostasis, it is unknown whether RSK2 plays a role in orthodontic tooth movement (OTM) and the development of OTM side effects such as external root resorptions. The reasons for the occurrence of these root resorptions are far from being understood, but recent studies suggest that genetics plays a decisive role in its development.^19–24^ For instance, loss of bone sialoprotein in mice caused extensive root resorptions due to defective mineralization of the acellular cementum layer.^25^ Moreover, other proteins expressed in the cementum layer were found to have protective effects against root resorption.^26^ The cementum layer is therefore regarded as a barrier against root resorption.^27^

Given the nonredundant role of RSK2 in bone and cementum formation we, therefore, asked how lack of RSK2 function in mice affects OTM and the emergence of root resorptions in vivo.

## Results

### Loss of *Rsk2* function accelerates tooth movement in mice

**Fig. 1:**
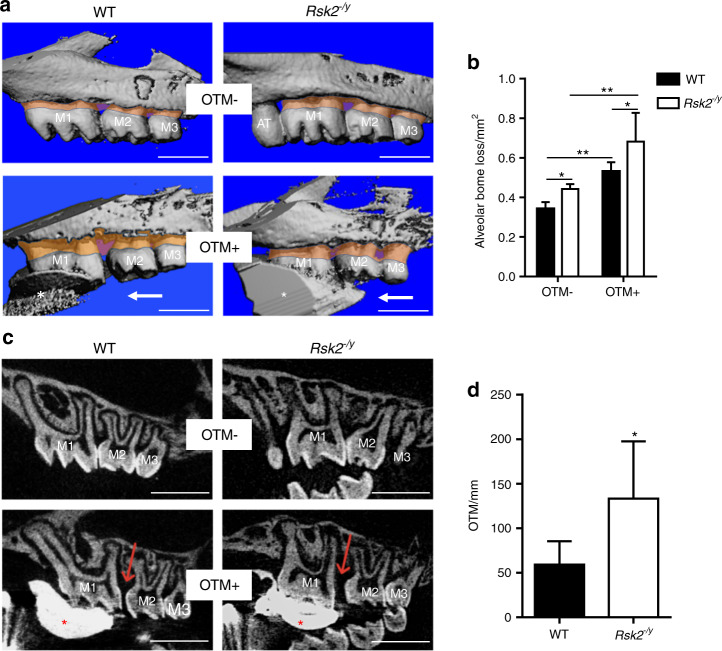
Loss of *Rsk2* function leads to faster tooth movement in mice. **a** Three-dimensional µ-CT reconstruction of maxillary teeth from 12 weeks old *Rsk2*^*−/y*^ mice and wild-type littermates. Upper panels show untreated teeth (OTM−) and lower panels show teeth after orthodontic tooth movement (OTM+). The orange area indicates the horizontal alveolar bone loss, measured from enamel–cementum-junction to the alveolar bone crest. White arrows indicate the direction of the applied force. The scanning artifacts caused by the light-curing resin (white asterisks) are outside the region of interests and do not affect the measurements. M1, first molar; M2, second molar; M3, third molar; AT, additional tooth; scale bars = 1 mm. **b** Quantification of the area of alveolar bone loss on the untreated (OTM−) and treated (OTM+) side in *Rsk2*^*−/y*^ (*n* = 4) and WT mice (*n* = 5). Values are means ± SD. (*P < 0.05, **P < 0.01) **c** µ-CT cross-sections through M1–M3 showing the approximal gap after orthodontic tooth movement. Scale bars = 1 mm. **d** Quantification of the intercoronal distance between M1 and M2 after 12 days of OTM in *Rsk2*^*−/y*^ and WT mice. *P < 0.05

We first analyzed the maxillary teeth of WT and *Rsk2*^−*/y*^ mice that were not subjected to OTM (OTM−) (Fig. 1a, upper panels). Our µCT analysis revealed that the exposed root area, a surrogate measure for alveolar bone loss, was significantly larger in *Rsk2*^*−/y*^ mice as compared to that of WT mice (Fig. 1b). This observation in combination with the existence of an additional tooth in some of the *Rsk2*^*−/y*^ mice confirmed our previous analysis of mandibular teeth from *Rsk2*^*−/y*^ mice.^18^ We next analyzed the alveolar bone of *Rsk2*^*−/y*^ and WT mice after OTM. We observed that OTM caused a loss of alveolar bone in *Rsk2*^*−/y*^ and WT mice (Fig. 1a, lower panels). This OTM-induced increase of alveolar bone loss was statistically significant in both groups. To determine the amount of OTM we next analyzed the crowns of the first and second molars on µCT cross-sections (Fig. 1c). The separation of the crowns after OTM was more apparent in *Rsk2*^*−/y*^ mice as compared to those of WT (Fig. 1c, lower panels). In fact, quantification of the gap between the two crowns revealed that OTM was more than two-fold faster in *Rsk2*^*−/y*^ mice (Fig. 1d).

### Periodontal response to OTM in *Rsk2*^*−/y*^ mice

**Fig. 2:**
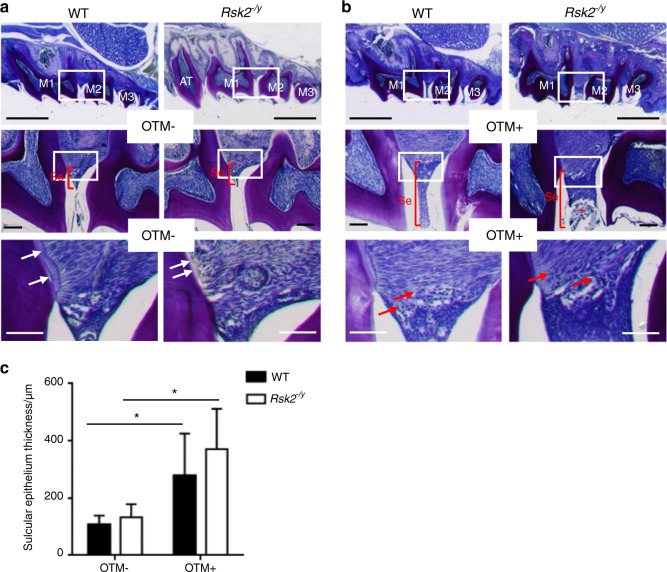
Histological analysis of tooth movement in *Rsk2*^−^^*/y*^ mice and wild-type mice. **a**, **b** Decalcified histological sections stained with toluidine blue of maxillary teeth from 12 weeks old *Rsk2*^*−/y*^ mice and WT mice. The areas indicated by the white boxes in the upper panels and middle panels are shown at higher magnifications in the middle and lower panels. The acellular cementum layer of teeth from *Rsk2*^−*/y*^ mice appears thinner as compared to those of WT mice (white arrows in **a**, lower panels). The OTM-induced approximal gap allows debris to accumulate (red asterisk in **b**, middle panels) causing inflammatory hyperplasia of the sulcular epithelium (se) with an influx of lymphocytes (red arrows in **b**, lower panels). Scale bars: upper panels are 1 mm; middle and lower panels are 100 µm. **c** Quantification of thickness of the sulcular epithelium in *Rsk2*^−*/y*^ and WT mice. Values are means ± SD. (*n* = 4–5). *P < 0.05

We next performed decalcified histology to analyze the periodontal response to OTM in *Rsk2*^*−/y*^ and WT mice (Fig. 2a, b). Our analysis of teeth without tooth movement (OTM−) revealed no clear differences between *Rsk2*^*−/y*^ and WT mice. Only the acellular cementum layer in *Rsk2*^−*/y*^ mice appeared thinner as compared to those of WT mice (Fig. 2a, lower panels). In line with the µCT evaluation we observed that the OTM-induced separation between the first molar (M1) and the second molar (M2) was wider in *Rsk2*^*−/y*^ mice as compared to that of WT (Fig. 2b). The OTM-induced gap between the teeth in *Rsk2*^*−/y*^ and WT mice caused food impaction and inflammatory hyperplasia of the sulcular epithelium associated with an accumulation of lymphocytes in the subepithelium (Fig. 2b, middle and lower panels). This edematous swelling is significantly increased in both groups after the treatment (Fig. 2c).

**Fig. 3:**
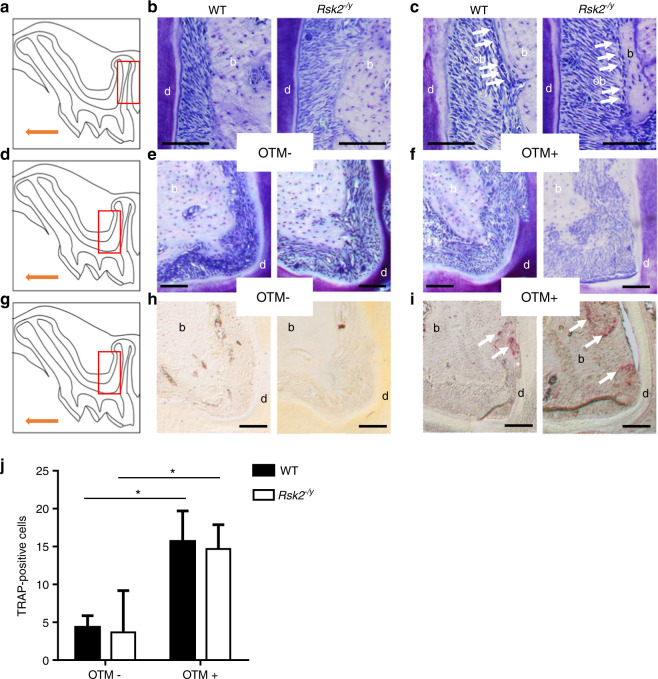
Histological evaluation of OTM-induced pressure and tension zones. **a**–**c** Schematic drawing of a murine, maxillary M1. The tension side (red box in **a**) was analyzed on toluidin-blue stained sections of maxillary teeth from 12 weeks old *Rsk2*^−^^*/y*^ and WT mice. White arrows indicate osteoblasts. **d**–**f** Toluidin-blue stained histological sections of the compression side from the same mice red box in **d**. Root resorption were evident after OTM (white asterisks). **g**–**i** Immunohistochemical TRAP-stained histological sections of the compression side from the same mice red box in **g**. Numerous TRAP-positive cells are evident after OTM in *Rsk2*^*−/y*^ and WT mice (white arrows in **i**). **j** Quantification of TRAP-positive osteoclasts in WT and *Rsk2*^*−/y*^ mice. Values are means ± SD. (*n* = 3). b, bone; d, dentin; ob, osteoblast. Scale bar = 100 μm. *P < 0.05

To further investigate the soft tissue response to OTM, we next analyzed zones of OTM-induced tissue pressure and tissue tension in *Rsk2*^−*/y*^ and WT mice (Fig. 3). We here focused on the distal root of the M1 where the periodontal ligament (PDL) is exposed to tension on the distal root surface (Fig. 3a–c) and on the mesial root surface to pressure (Fig. 3d–f). Our analysis of untreated teeth revealed that most of the alveolar bone in *Rsk2*^*−/y*^ and WT mice was covered by bone lining cells and only a few active osteoblasts were evident (Fig. 3b). In contrast, after OTM we observed on the tension side multiple active, secreting osteoblasts on the alveolar bone surface in both *Rsk2*^*−/y*^ and WT mice. The observation of cement lines further indicated OTM-induced bone formation. Conversely, our histological analysis of the pressure side revealed signs of bone resorption after OTM as indicated by the presence of osteoclasts in *Rsk2*^*−/y*^ and WT mice (Fig. 3f). To better visualize these osteoclasts, we performed a staining with tartrate-resistant acid phosphatase (TRAP). Whereas no osteoclasts were evident in the PDL of teeth that were not subjected to tooth movement (Fig. 3h), multiple TRAP-positive cells were found in the pressure zones of teeth from *Rsk2*^*−/y*^ and WT mice after OTM. These TRAP-positive cells were evident not only on the alveolar bone surface but also within the PDL and on the root surface near root resorptions (Fig. 3i). Quantification of TRAP-positive cells showed a significant increase in both groups after OTM (Fig. 3j). Taken together, our histological analysis revealed that OTM induces a characteristic periodontal response in *Rsk2*^*−/y*^ mice that does not seem to differ from those of WT mice.

### Cementum hypoplasia does not increase the amount of OTM-induced root resorption in *Rsk2*^*−/y*^ mice

**Fig. 4:**
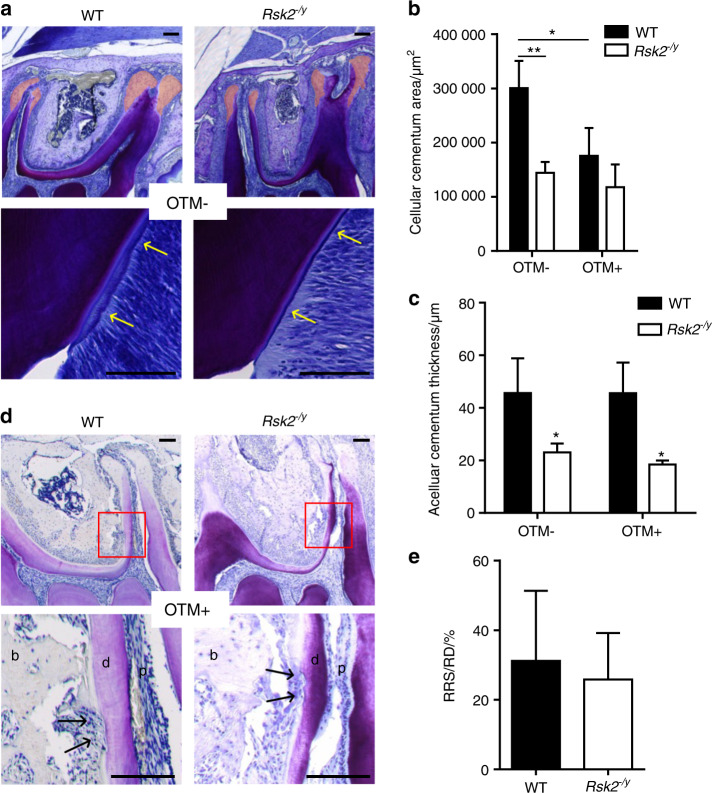
Histological examination of the cementum layer and OTM-induced root resorptions. **a** Decalcified histological sections stained with toluidine blue of maxillary teeth from 12 weeks old *Rsk2*^*−/y*^ mice and WT mice. Orange area indicates cellular cementum and yellow arrows indicate acellular cementum. **b** Quantification of the area of cellular cementum and the thickness of acellular cementum in **c** in *Rsk2*^*−/y*^ and WT mice. Values are means ± SD. (*n* = 3–5, *P < 0.05, **P < 0.01). **d** Toluidine blue stained histological sections of the same mice showing root resorptions. The lower panels are magnifications of the areas depicted by the red rectangles in the upper panels. Black asterisks indicate root resorptions. b, bone; d, dentin; p, pulp. **e** Quantification of the resorbed root surface per root surface (RRS/RS) of 12 weeks old *Rsk2*^*−/y*^ (*n* = 4) and WT mice (*n* = 5). Scale bar = 100 µm. Values are means ± SD

We finally determined whether the loss of Rsk2 function may increase the risk of OTM-induced root resorption. We, therefore, first quantified cellular and acellular cementum of teeth that were not subjected to tooth movement in *Rsk2*^*−/y*^ and WT mice (Fig. 4a). Confirming our previous observations on mandibular teeth of *Rsk2*^−*/y*^ mice,^18^ we observed a significant reduction of cellular cementum area and acellular cementum thickness in *Rsk2*^*−/y*^ mice (Fig. 4b, c). Interestingly, we also found a significant reduction of the cellular cementum area after OTM in WT teeth (Fig. 4b). This OTM-induced reduction of cellular cementum was not observed in *Rsk2*^*−/y*^ mice. Although the overall thickness of acellular cementum was not affected by OTM in WT and *Rsk2*^*−/y*^ mice (Fig. 4c), the cementum layer was partially lost and resorption lacunae were evident (Fig. 4d). These resorptions extended in the dentin layer and were thus identified as lateral root resorption. The shape and the localization of these resorptions did not differ between WT and *Rsk2*^*−/y*^ mice. Our quantification of the percentage of root resorption per root surface on the compression side also revealed no significant differences between WT and *Rsk2*^*−/y*^ mice (Fig. 4e). Taken together, these results indicate that cementum hypoplasia in *Rsk2*^*−/y*^ mice does not increase the amount of OTM-induced root resorption.

## Discussion

Our study confirms that RSK2 has a decisive influence on the structures of the periodontium and demonstrates, for the first time, that RSK2 plays an important role in OTM.

The typical dental finding diagnostic of CLS in children is the premature loss of teeth.^14–17,28^ This is partially phenocopied in *Rsk2*-deficient mice, which show an increased loss of alveolar bone.^18^ In this study, we also observed this horizontal alveolar bone loss around maxillary molars that were not subjected to tooth movement. However, it is unlikely that the reason for the alveolar bone loss in *Rsk2*^*−/y*^ mice is a systemic defect of osteoblasts as we used rather young (10–12 weeks old) mice for this study and the osteopenic phenotype of *Rsk2*^−*/y*^ mice develops at an older age.^3^ This was also documented by our µ-CT analysis of calvarial and palatal bone where we did not observe any significant differences between *Rsk2*^−/*y*^ mice and WT littermates (Supplementary Fig. 1). Furthermore, quantitative backscattered electron imaging revealed normal mineralization of trabecular bone in *Rsk2*^−/*y*^ mice (Supplementary Fig. 2). This indicates that trabecular bone morphology and quantity is not significantly affected by loss of RSK2 function in young mice. It is, therefore, more likely that the alveolar bone loss is caused by a cell-autonomous defect of the cementoblasts leading to dysfunctional acellular cementum formation as described previously.^18^

Accordingly, our histological analysis of maxillary molars revealed a reduced thickness of acellular cementum in *Rsk2*^*−/y*^ mice. The periodontal phenotype of *Rsk2*^*−/y*^ mice, therefore, seems to be comparable to that of BSP-deficient mice, in which hypoplasia of acellular cementum leads to periodontal dysfunction and subsequent to a reduced ability to resist mechanical forces, leading to alveolar bone loss.^25,29–31^

It is also certainly possible that RSK2, as a member of the MAPK pathway, affects signal transduction pathways during OTM. Indeed, several studies have demonstrated that the MAPK pathway plays an important role during OTM.^32,33^ For instance, it has been shown that stress applied to PDL cells activates downstream targets of the MAPK pathways such as ERK1/2 and p38. Interestingly, inhibition of these target genes can also decrease the expression of osteoblast markers such as ALP, OPN, and BSP. It is, therefore, possible that lack of RSK2 also causes a reduction of ALP, OPN, and BSP during OTM, as RSK2 acts as a downstream kinase of these pathways. Moreover, it is noteworthy that mechanical stimulation of human periodontal fibroblasts induced the expression of the RSK2 substrate ATF4, which acts as an important factor for osteoblast differentiation from PDL cells.^34^ Whereas our study cannot clarify the exact biological role of RSK2 during OTM, we clearly demonstrated that RSK2 does affect OTM in vivo.

Besides accelerated tooth movement we also observed that orthodontic forces in *Rsk2*^*−/y*^ mice significantly exacerbated the alveolar bone loss.^31^ Interestingly, dysfunctional cementum function in BSP-deficient mice also reduces the ability of the PDL to resist forces and leads to mechanical-induced further alveolar bone loss.^31^ However, this seems unlikely to be the case in *Rsk2*^*−/y*^ mice as the amount of OTM-induced bone loss was not higher than those of control animals. We rather believe that debris niches due to the separation of the molars and the orthodontic appliance cause periodontitis and periodontal breakdown. In fact, our histological analysis of teeth after OTM revealed in both *Rsk2*^*−/y*^ and WT mice clear signs of gingival inflammation apparent as significant swelling of sulcular epithelium and subepithelial lymphocyte influx. Another reason for the bone loss after OTM could be the application of supraphysiological forces. However, Taddei et al. described that the force of 0.35 N, which we used in our study, is necessary to obtain tooth movement and causes relatively little tissue damage in mice.^35^ Our histological analysis indicated that the force level was not too high as we observed a normal cellular response to OTM in *Rsk2*^*−/y*^ and WT mice. This cellular response did not seem to differ between *Rsk2*^*-/y*^ and WT mice. In fact, both groups showed a significant increase in TRAP-positive osteoclasts after OTM, a distinct sign of bone remodeling.

Another important histological finding was that only a few areas of pressure-induced tissue necrosis (i.e., hyalinization) were evident in *Rsk2*^−*/y*^ and WT (data not shown). Small areas of hyalinization are almost unavoidable during OTM^31,36^ and only large areas of hyalinization would have been indicative for excessive forces. Also, the presence of root resorption in *Rsk2*^*−/y*^ and WT mice should be considered as a normal histological sign of OTM.^37^

The reasons for the occurrence of orthodontic root resorption are far from understood but recent studies suggest that genetics plays a decisive role in its development.^25–27,29,30^ In this regard, it was of particular interest for us to analyze the role of RSK2 in the development of root resorption. Since acellular cementum has a protective function against lateral root resorptions,^25–27,38^ we assumed that a reduced layer of acellular cementum in *Rsk2*^*−/y*^ mice leads to more root resorptions;^18^ however, this could not be confirmed in our study. No significant differences were observed between *Rsk2*^*−/y*^ mice and WT mice with regard to the percentage of resorptions in relation to the root surface, or to the shape and localization of the resorptions. It, therefore, seems that also a reduced layer of acellular cementum may protect against root resorption as long as the integrity of the cementum layer is preserved. Another reason for the small amount of root resorption in *Rsk2*^*−/y*^ mice could be the alveolar bone phenotype.^26,39–41^ In fact, as root resorptions occur more frequently in areas of high tissue pressure^40,41^ it is possible that the alveolar bone loss of *Rsk2*^*−/y*^ mice causes less resistance to OTM. This does not only seem to accelerate OTM but may also decrease the risk of root resorptions. Nevertheless, we can conclude that loss of RSK2 function is not a risk factor for the development of OTM-induced root resorptions. This does not imply that loss of RSK2 function may not affect the repair of root resorptions, which seems to be governed by the same molecular mechanism that also controls cementogenesis during tooth development.^20,21,39,41,42^ Since loss of RSK2 function negatively affects cementogenesis, the repair of root resorptions may be impaired in *Rsk2*^*−/y*^ mice. This should be addressed in futures studies.

The unique dental phenotype of *Rsk2*^*−/y*^ mice caused some limitations in the experimental setup. The presence of small, supernumerary teeth mesial to the M1 in *Rsk2*^−/*y*^ mice was striking and confirmed previous observations.^43,44^ It seems that the loss of RSK2 function leads to the reappearance of diastemal teeth, a type of tooth that was lost in rodents during evolution.^43^ As these diastemal teeth may affect tooth movement it was important to exclude mice showing bilateral a supernumerary tooth in the upper dentition from our experiments. The number of animals per group in our study is therefore comparatively low and it is possible that more significant differences would have been found had more mice been used.

Despite these difficulties, our results demonstrate that our orthodontic mouse model is appropriate for analyzing the role of individual genes during OTM and the development of root resorptions in vivo.

## Conclusion

Taken together, we can conclude that RSK2 plays a nonredundant role in OTM. Loss of *Rsk2* function accelerates tooth movement without causing more root resorptions, although *Rsk2*^−/*y*^ mice exhibit reduced levels of acellular cementum. We, therefore, believe that the relationship between root cementum and orthodontic root resorption is less clear than previously expected. Future studies should use transgenic mice to decipher the genetic interplay between cementogenesis and orthodontic root resorptions.

## Materials and methods

### Mice

*Rsk2*-deficient mice (*Rsk2*^−*/y*^)^3,4^ and control wild-type littermates (WT) were maintained at the animal facility of the University Medical Center Hamburg–Eppendorf on a C57BL/6J background. The generation of *Rsk2*^*−/y*^ mice by homologous recombination has been described previously.^4^ Briefly, the targeting vector was located in exon 2 of the *Rsk2* gene (*Rps6ka3*) and contained a neomycin resistance gene, followed by three stop codons and flanked by two loxP sites. The construct was electroporated in ES cells and, after NeoR selection, injected into C57BL/6J blastocysts. The NeoR cassette was finally removed by crossing with a C57BL/6J/CMV-Cre transgenic line. *Rsk2*^*−/y*^ mice are viable and show reduced body weight (Supplementary Fig. 3). The bone phenotype of *Rsk2*^*−/y*^ mice was the subject of several studies and is characterized by osteopenia due to osteoblast dysfunction.^3,4,45^ *Rsk2*^*−/y*^ mice additionally exhibit craniofacial dysmorphia and alveolar bone loss due to cementum defects.^18,43^ Due to the x-chromosomal inheritance of the *Rsk2* gene only male animals were used in this study. All experiments were carried out on 4–5 mice per genotype at 10 weeks of age at the time of the orthodontic procedure. The weight of each animal was measured over the entire period of the experiments. In both groups there was a slight, but not significant, reduction of body weight in the first three days after anesthesia (Supplementary Fig. 3). The animals were subjected to a normal 12 h light/dark rhythm at a constant temperature of 24 °C. Furthermore, they were kept on a soft food diet consisting of mashed dry food pellets to prevent mechanical damage to the orthodontic appliance. All animal procedures were performed under consent with the commission for animal welfare (Behörde für Gesundheit und Verbraucherschutz der Hansestadt Hamburg, Nr. 072/18).

### Orthodontic appliance

The experiment was carried out as a split-mouth model in which the untreated side of the jaw served as an internal control.^46^ The mice were anesthetized with a solution of ketamine (40 mg· kg^−1^ bw Ketamin-S), xylazine 2% (16 mg· kg^−1^ bw), and heparin (40 000 IE per kg bw in 0.9% NaCl) (10 mL· kg^−1^). OTM was not performed in *Rsk2*-deficient mice that showed an additional tooth mesial to the M1.^17,43^ The anesthetized mice were carefully fixed on a treatment lathe (Unimat 3, Emco, Wiener Neudorf, Austria). The maxillary incisors were then attached to the operating table with a loop of suture material (Vicryl, Ethion Inc., New Jersey, USA) (Fig. 1a), and the incisors of the mandible were fixed with an orthodontic rubber chain (Elasto-force, Dentaurum, Ispringen, Germany). All procedures were performed using magnifying glasses (Zeiss G 2.5 TTL, Carl Zeiss Germany, Oberkochen, Germany) and a headlamp (Sigma Smart study, Sigma Dental Systems, Handewitt, Germany). The surface of the left first maxillary molar and incisors were etched with 37% phosphoric acid gel (HS-etch gel 37%, Henry Schein Dental, Langen, Germany). The bonding (Scotchbond, 3 m Espe, Neuss, Germany) was applied after removal of the etching gel and sufficient drying of the surface. The distal end of the Nitinol open coil spring (Sentalloy Open Coil Spring, Dentsply Sirona, Pennsylvania, USA) was bonded to the surface of the left first maxillary molar with a light-cured resin (Estelite Flow Quick, Tokuyama Dental Corp., Tokyo, Japan). The treatment table was moved backward, parallel to the direction of the force, to achieve the desired activation of 35 centinewton that was controlled with a tension gauge (tension gauge, Dentaurum, Ispringen, Germany). With achieving the desired force the anterior part of the coil spring was then bonded to both incisors (Fig. 1a, b). The animals were monitored on a warming mat after the orthodontic procedure until they woke up from anesthesia. After 12 days of OTM, the animals were euthanized by CO_2_ inhalation. The heads were fixed for 24 h in formalin (Formafix 3,5%, Grimm med Logistik GmbH, Torgelow, Germany) and then transferred to 80% ethanol.

### Contact radiography and micro-computed tomography imaging

The skulls were radiographed in a digital contact radiography cabinet (Faxitron X-ray Corp., Wheeling, IL, USA) (Fig. 1c) and scanned with a voxel size of 15 µm. Micro-CT images (µCT 40 Scanco Medical, Bassersdorf, Switzerland) were acquired at 55 kVp with an anode current of 145 µA and an integration time of 200 ms. The reconstruction of the raw data images was performed with the scanco µCT evaluation program V6.6. Analysis of µCT images was performed using ImageJ 1.52 (National Institutes of Health, Bethesda, MD, USA) and its implication BoneJ 1.4.3.^47^ The area of bone loss was pseudo-colored with Photoshop Cs4 (Adobe Systems Inc., Mountain View, CA, USA).

### Histology and histomorphometry

After performed µCT-analysis the upper jaws were prepared for histological examination. They were decalcified using Usedcalc (MEDITE Medical GmbH, Burgdorf, Germany), an EDTA containing reagent, for 21 days, while the reagent was changed once a week. Afterward, the upper jaws were cut in the parasagittal plane. The halves were dehydrated in ascending alcohol concentrations and embedded in paraffin. Sections of 4 µm thickness were cut using a microtome (Supercut 2050, Reichert-Jung, Leica Microsystems GmbH, Wetzlar, Germany) and stained with toluidine blue. For this, the sections were deparaffined in xylene baths (3 × 5 min). The samples were rehydrated in descending alcohol series for 2 min each, followed by a short wash in distilled water. Afterward, they were dyed in 1% toluidine blue (pH 4.5) for 30 min. The stained sections were then rinsed with distilled water and in the next step with 80% ethanol, followed again by a short wash in distilled water, in order to dehydrate them in an ascending alcohol series. Before covering with Eukitt (ORSAtec GmbH, Bobingen, Germany), an infiltration with xylene was executed in three consecutive baths for 5 min each. TRAP staining was performed as follows: the sections were dewaxed in xylene (3 × 5 min) and rehydrated in a descending alcohol series as described for the toluidine blue staining. Totally, 50 mL buffer solution is prepared from 40 mmol·L^−1^ sodium acetate (pH 5) and 10 mmol·L^−1^ sodium tartrate. This is mixed with 5 mg Naphthol-AS-MX phosphate dissolved in 500 μL dimethylformamide. Then 30 mg Fast Red Violet is added and homogenized while shaking. The prepared sections get immersed in the solution for about 120 min and incubated at 37 °C. The samples were not counterstained and covered with Aquatex (Merck KGaA, Darmstadt, Germany) in the end.

All histomorphometric analyses were done using the Osteo-Measure histomorphometry system (Osteometrics, Atlanta, GA, USA). Cellular cementum was quantified at both root apices of the first maxillary molar and the thickness of acellular cementum was quantified along its full length on the distal root surface of the first maxillary molar as previously described.^18^

### Quantitative backscattered electron imaging (qBEI)

**Fig. 5:**
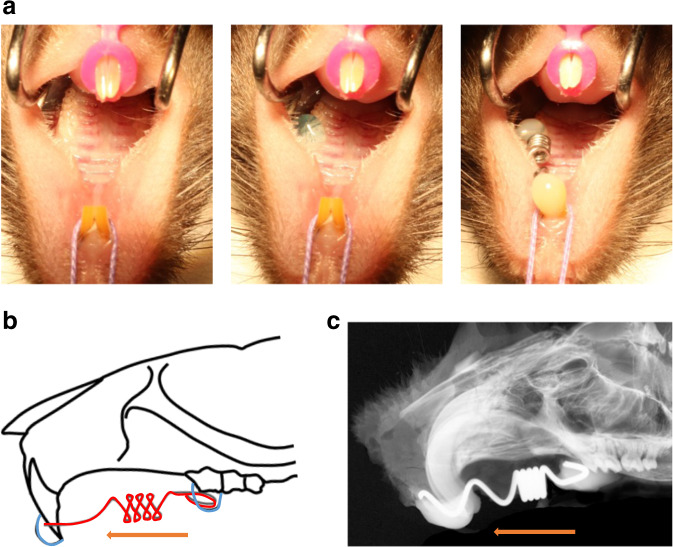
Description of the experimental setup. **a** Photographs of the procedure. The upper and lower incisors are attached to the table using a suture and rubber chain (left picture). The teeth were etched with 37% phosphoric acid gel (middle picture) and the coil spring was then fixed with light-curing resin to the first molar and the incisors (right picture). **b** Illustration of the OTM model and contact radiography of the appliance in **c**. The NiTi-tension spring (red) was attached to the incisor and the first molar using a dental light-curing resin (blue). The orange arrows indicate the mesial direction of the force

Methylmethacrylate-embedded specimens of vertebrae from 12-week-old WT and *Rsk2*^−/*y*^ mice were polished and carbon-coated. Afterward, they were mounted in a scanning electron microscope (LEO 435 VP; LEO Electron Microscopy Ltd., Cambridge, England) with a backscattered electron detector (Type 202, K.E. Developments Ltd., Cambridge, England). To determine the mineralization of the specimens they were scanned at 20 kV and a 680-pA electron beam current. Images were acquired at 20× and 150× magnification and analyzed using a customized MATLAB (TheMathWorks, Inc. Natick, Massachusetts, USA) script. Generated gray values were transferred in the mean calcium content. For each specimen the mean calcium content (Ca Mean) and the mineralization heterogeneity (Ca Width) were analyzed. In each individual, three images per vertebrae were considered for the qBEI evaluations (Fig. 5).

### Statistical analysis

The statistical analysis was performed with GraphPad PRISM6 (GraphPad Software, San Diego, USA). We analyzed the data for normal distribution using the Shapiro–Wilk test. Results were expressed as the mean ± SD. Two-sided *t* test and one-way ANOVA with Bonferroni post hoc test were used for multiple group comparisons. *P* values below 0.05 were considered statistically significant.

## Supplementary information

Suppl. Figure 1, Suppl. Figure 2, Suppl. Figure 3
