# Supplementary material for: Accelerated tooth movement in Rsk2-deficient mice with impaired cementum formation
Source: Int J Oral Sci. 2020 Dec 23;12:35. doi: 10.1038/s41368-020-00102-4 (PMC7755898; doi:10.1038/s41368-020-00102-4)
Supplement: Supplementary file 1 — Suppl. Figure 1, Suppl. Figure 2, Suppl. Figure 3 [file 41368_2020_102_MOESM1_ESM.pdf]

Suppl. Figure 1

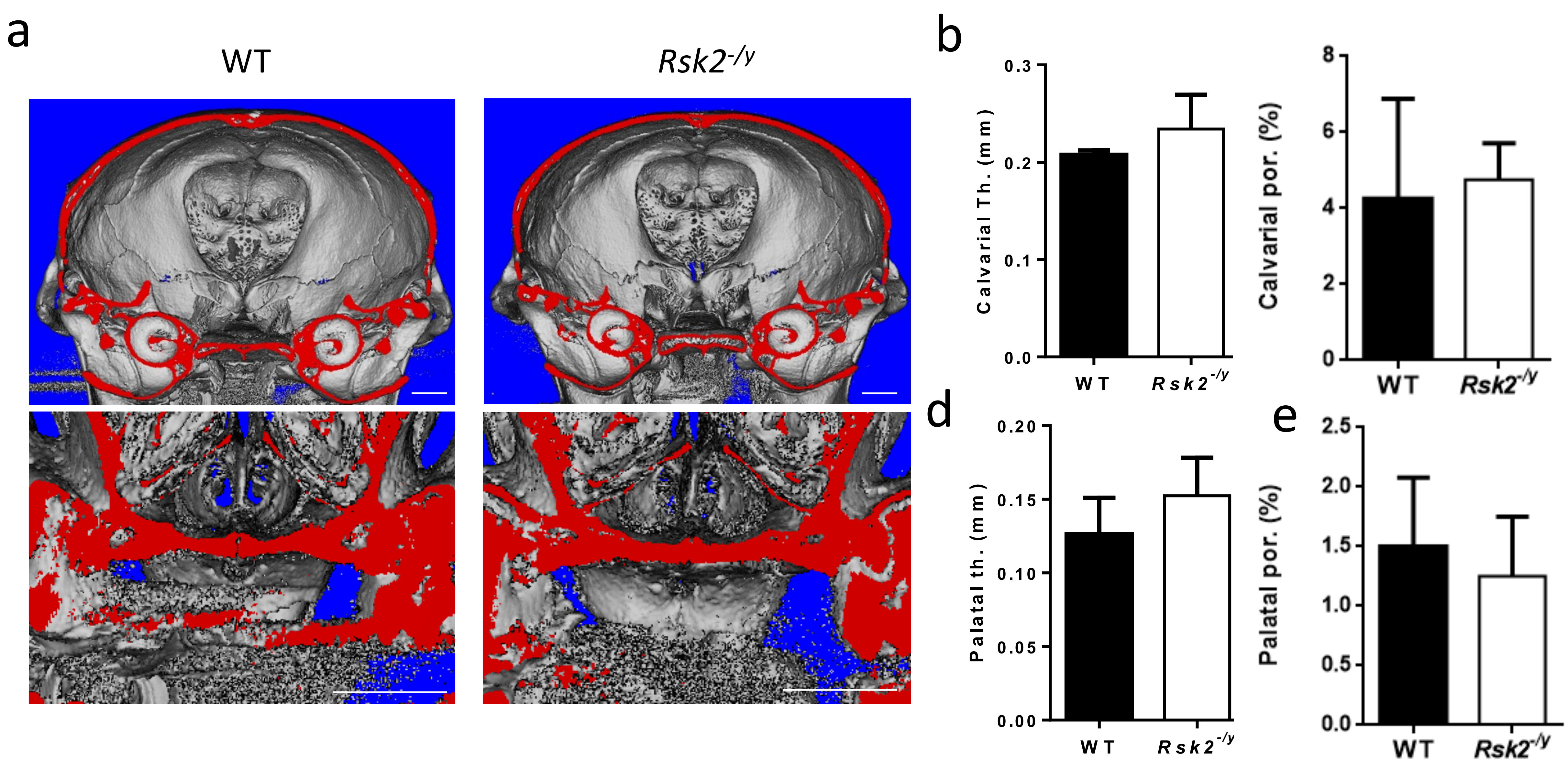

Suppl. Fig. 1. Normal calvarial and palatal bone in *Rsk2*$^{-/y}$ mice. (a) Three-dimensional µCT reconstruction of calvaria and palate of 12-week-old *Rsk2*$^{-/y}$ mice and wildtype littermates. **(b-e)** Calvarial thickness (b), calvarial porosity (c), palatal thickness (d), and palatal porosity (e) were quantified. n= 4. Values are means ± SD *P <0.05 Scale bars = 1mm.

Suppl. Figure 2

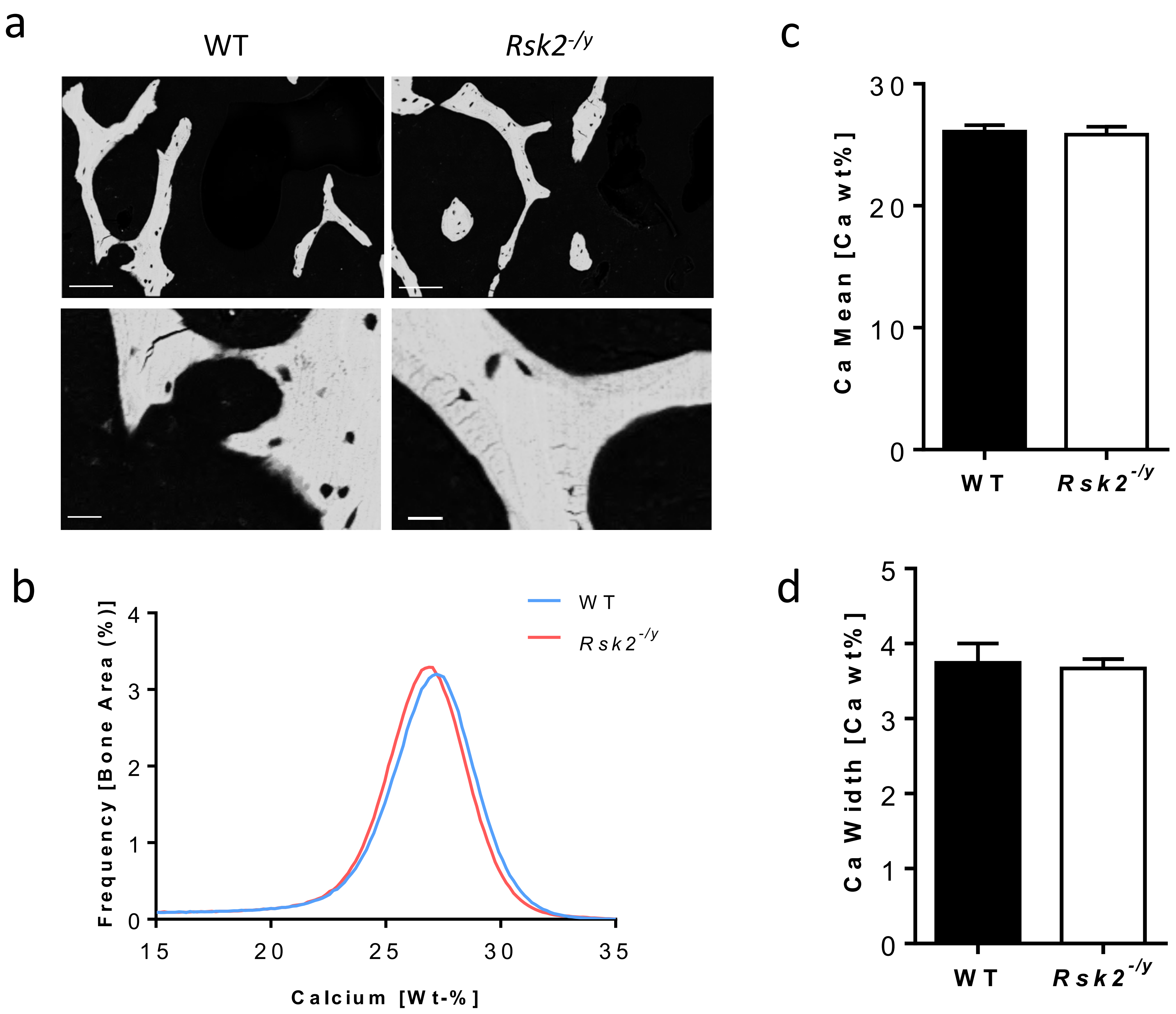

Suppl. Fig. 2. Bone mineral density distribution (BMDD) of trabeceluar bone in *Rsk2*$^{-/y}$ mice (a) Quantitative backscattered electron imaging (qBEI) of vertebrae from 12-week-old *Rsk2*$^{-/y}$ mice and wildtype littermates. Images show the calcium content of single bone trabeculae. Scale bar = 20µm (upper panels) and 100µm (lower panels). **(b)** Histograms of the qBEI images showing the calcium distribution, **(c-d)** Quantification of the mean mineral content (Ca mean) and the heterogeneity of the calcium distribution (Ca width). n=4. Values are means ± SD.

Suppl. Fig. 3

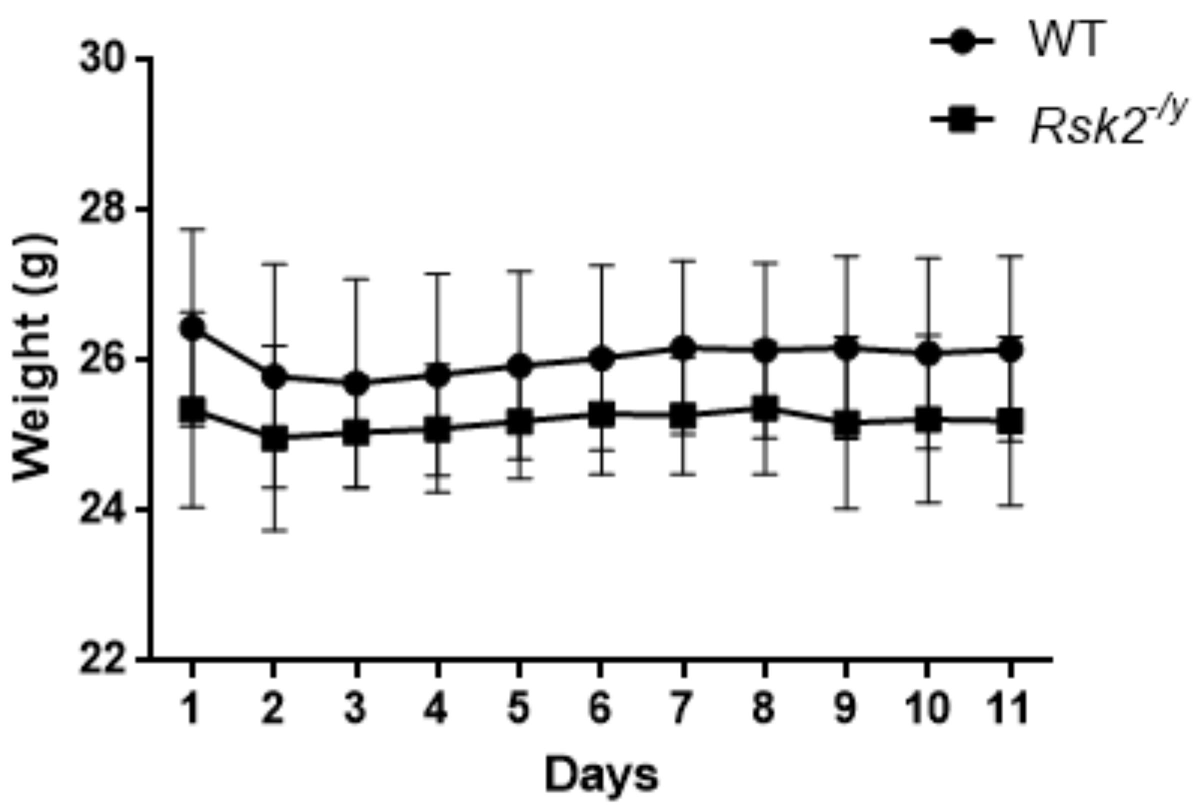

Suppl. Fig. 3. Weight curve of WT and Rsk2-/y mice during the time of orthodontic treatment. (Values are means ± SD)
